# Supplementary material for: Diversity and Ecology of Lobophora Species Associated with Coral Reef Systems in the Western Gulf of Thailand, including the Description of Two New Species
Source: Plants (Basel). 2022 Dec 2;11(23):3349. doi: 10.3390/plants11233349 (PMC9739394; doi:10.3390/plants11233349)

Figure S1. Specimen-level maximum likelihood (PhyML) phylogenetic tree of the brown algal genus *Lobophora* (Dictyotlaes, Phaeophyceae) constructed using the mitochondrial *cox*3 sequence (724 bp). Branch support values (aLRT) are displayed at the nodes.

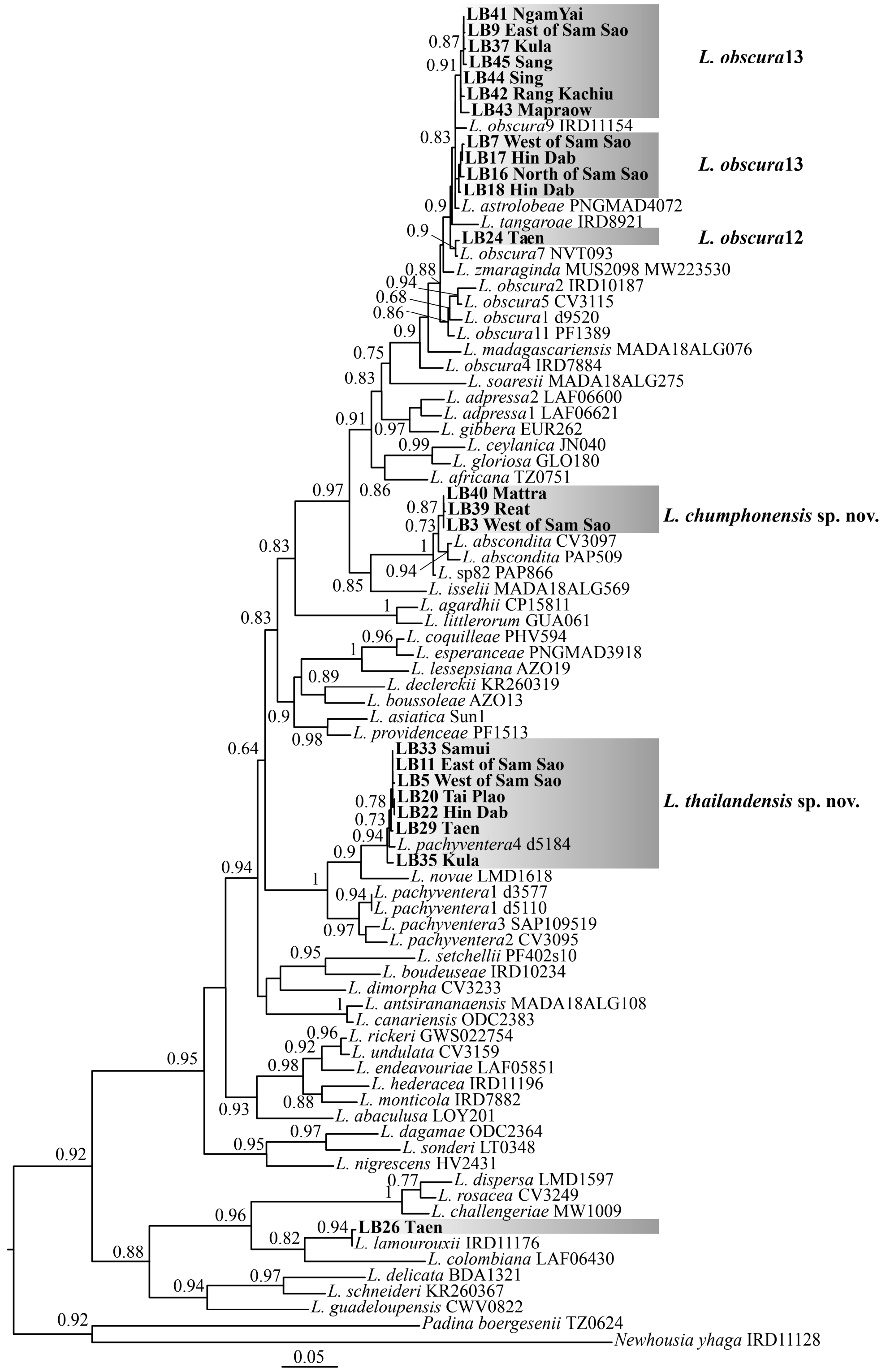

Supplement: Supplementary file 1 [file plants-11-03349-s001.zip › Figure S1. Specimen-level maximum likelihood (PhyML) phylogenetic tree of the brown algal genus Lobophora (Dictyotlaes, Phaeophyceae) construc.pdf]
