# Supplementary material for: Diversity and Ecology of Lobophora Species Associated with Coral Reef Systems in the Western Gulf of Thailand, including the Description of Two New Species
Source: Plants (Basel). 2022 Dec 2;11(23):3349. doi: 10.3390/plants11233349 (PMC9739394; doi:10.3390/plants11233349)
Supplement: Supplementary file 1 [file plants-11-03349-s001.zip › Figure S4. In situ photographs of interaction between Lobophora and corals.pdf]

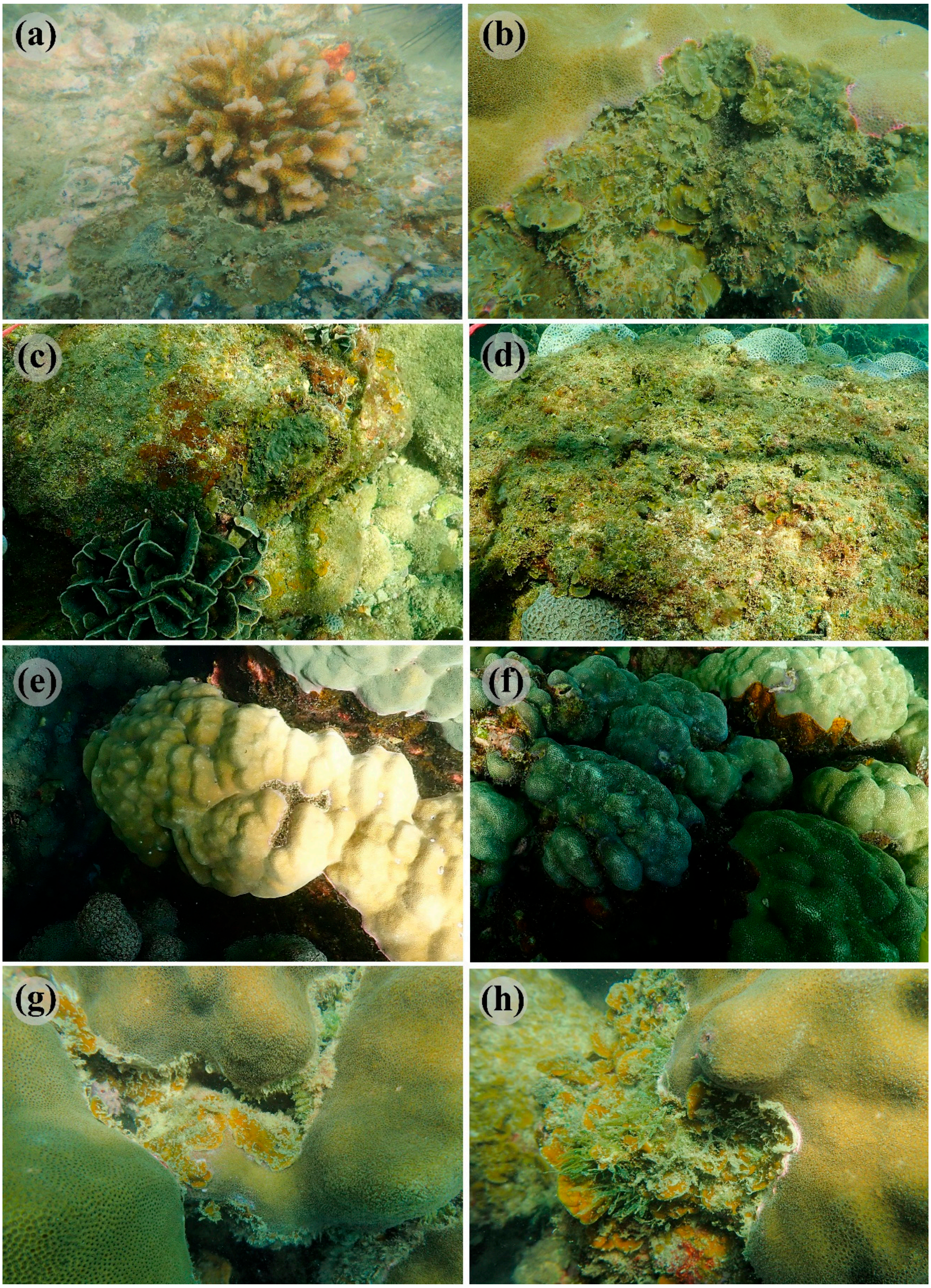

Figure S4. *In situ* photographs of interaction between *Lobophora* and corals; *L. obscura*13# (a,b,c,d), *Lobophora chumphonensis* sp. nov. (e,f), and *Lobophora thailandensis* sp. nov. (g,h)
