# Supplementary material for: Diversity and Ecology of Lobophora Species Associated with Coral Reef Systems in the Western Gulf of Thailand, including the Description of Two New Species
Source: Plants (Basel). 2022 Dec 2;11(23):3349. doi: 10.3390/plants11233349 (PMC9739394; doi:10.3390/plants11233349)
Supplement: Supplementary file 1 [file plants-11-03349-s001.zip › Table S1. Interactions between Lobophora species and scleractinian corals in the study sites.pdf]

Table S1. Interactions between *Lobophora* species and scleractinian corals in the study sites

Corals	Algal species				
	L. lamourouxii	*L. obscura*12	*L. obscura*13	*L. chumphonensis* sp. nov.‡	*L. thailandensis* sp. nov.
Diploastrea spp.	0	0	0	0[a]<br>1.39[b]	1.16[a]<br>0[b]
Favia spp.	0	0	2.60[a]<br>7.79[b]	0	2.35[a]<br>3.53[b]
Favites spp.	0	0	9.89[a]<br>17.37[b]	0	4.59[a]<br>10.59[b]
Goniopora spp.	0	0	0[a]<br>1.29[b]	0	0
Goniastrea spp.	0	0	3.89[a]<br>1.29[b]	0	0
Pavona spp.	0	0	0[a]<br>12.99[b]	0	0[a]<br>7.06[b]
Pocillopora spp.	0	0	0[a]<br>2.60[b]	0	0[a]<br>17.65[b]
Porites spp.	0	0	12.99[a]<br>3.89[b]	78.33[a]<br>10.55[b]	18.94[a]<br>5.88[b]
Non-coral substrate[c]	100	100	23.41	9.72	28.25

[a] growing on live corals, [b] growing at the base of live corals or vicinity of live corals included dead corals, [c] growing on seaweed bed, coral rubble and bedrock
