# Supplementary material for: Diversity and Ecology of Lobophora Species Associated with Coral Reef Systems in the Western Gulf of Thailand, including the Description of Two New Species
Source: Plants (Basel). 2022 Dec 2;11(23):3349. doi: 10.3390/plants11233349 (PMC9739394; doi:10.3390/plants11233349)
Supplement: Supplementary file 1 [file plants-11-03349-s001.zip › Table S3. Primers, PCR and sequencing conditions.pdf]

Table S3. Primers, PCR and sequencing conditions used in this study. Temperature (Temp.) is shown in Celsius degrees, and time in minutes.

Primer	Direction	Sequence (5′→3′)	Initial Denaturation		Denaturation		Annealing		Elongation		Final Elongation	
			Temp.	Time	Temp.	Time	Temp.	Time	Temp.	Time	Temp.	Time
*cox*3-D1 F	F	GTDGAYCCNAGYCCDTGGCC	94	3	94	1	46	1	72	2	72	10
*cox*3-P2 R	R	ACAAARTGCCAATACCAAGC	94	3	94	1	46	1	72	2	72	10
*psb*A-F	F	ATGACTGCTACTTTAGAAAGACG	94	1	94	0.45	42	30	72	0.45	72	7
*psb*A-R	R	GCTAAATCTARWGGGAAGTTGTG	94	1	94	0.45	42	30	72	0.45	72	7
*rbc*L 68F	F	TGCCWAAATGGGRWAYTGGGATGC	94	3	94	0.45	53	0.45	72	1	72	6
*rbc*L 708R	R	TTAAGNTAWGAACCYTTAACTTC	94	3	94	0.45	53	0.45	72	1	72	6
*rbc*L 543F	F	CCWAAATTAGGTCTTTCWGGWAAAAA	94	3	94	0.45	53	0.45	72	1	72	6
*rbc*L 1391R	R	TCNAANGTAATATCTTTCCATA	94	3	94	0.45	53	0.45	72	1	72	6

All primer in this study was published by Draisma et al. (2001), Bittner et al. (2008), Ni-Ni-Win et al. (2008), Sun et al. (2012).

References

Bittner, L., Payri, C., Couloux, A., Cruaud, C., De Reviers, B. and Rousseau, F. 2008. Molecular phylogeny of the Dictyotales and their position within the Phaeophyceae, based on nuclear, plastid and mitochondrial DNA sequence data. Mol. Phylogenet. Evol. **49**: 211–226.

Draisma, S. G., Prud'homme van Reine, W. F., Stam, W. T. and Olsen, J. L. 2001. A reassessment of phylogenetic relationships within the Phaeophyceae based on RUBISCO large subunit and ribosomal DNA sequences. J. Phycol. **37**: 586–603.

Ni-Ni-Win, Hanyuda, T., Arai, S., Uchimura, M., Aboott, I. A. and Kawi, H. 2008. Three new records of Padina in Japan based on morphological and molecular markers. Phycol. Res. **56**: 288–300.

Sun, Z., Hanyuda, T., Lim, P.E., Tanaka, J., Gurgel, C. F. D. and Kawai, H. 2012. Taxonomic revision of the genus *Lobophora* (Dictyotales, Phaeophyceae) based on morphological evidence and analyses *rbc*L and *cox*3 gene sequences. Phycologia **51(5)**: 500–512.
