# Supplementary material for: Diversity and Ecology of Lobophora Species Associated with Coral Reef Systems in the Western Gulf of Thailand, including the Description of Two New Species
Source: Plants (Basel). 2022 Dec 2;11(23):3349. doi: 10.3390/plants11233349 (PMC9739394; doi:10.3390/plants11233349)
Supplement: Supplementary file 1 [file plants-11-03349-s001.zip › Table S5. Distance matrix of Lobophora species with euclidean method.pdf]

Table S5 Distance matrix of *Lobophora* species with euclidean method

	**LL (1)**	**LL (2)**	**LL (3)**	**LO12 (1)**	**LO12 (2)**	**LO12 (13)**	**LO13 (1)**	**LO13 (2)**	**LO13 (3)**	**LC (1)**	**LC (2)**	**LC (3)**	**LT (1)**	**LT (2)**
**LL (2)**	0.09543													
**LL (3)**	0.07848	0.11448												
**LO12 (1)**	1.62575	1.60061	1.66862											
**LO12 (2)**	2.99947	2.97320	3.04878	1.39949										
**LO12 (3)**	1.53202	1.51120	1.57314	0.13796	1.51383									
**LO13 (1)**	3.01380	2.99705	3.05005	2.32458	2.48857	2.35505								
**LO13 (2)**	2.98379	2.96835	3.01835	2.30540	2.50003	2.33086	0.10792							
**LO13 (3)**	2.18649	2.16619	2.23439	1.22666	1.62006	1.27286	1.13877	1.14037						
**LC (1)**	4.47200	4.48417	4.43565	4.41781	5.09103	4.33312	4.26700	4.16091	4.38925					
**LC (2)**	3.42901	3.44307	3.40181	3.44745	4.22575	3.36270	3.09805	2.99192	3.25567	1.30847				
**LC (3)**	4.70006	4.71387	4.67274	4.42660	4.93114	4.35135	4.22929	4.12379	4.36813	0.68978	1.57029			
**LT (1)**	1.19370	1.19059	1.21453	1.69693	2.85857	1.63283	1.96937	1.93312	1.49169	4.03828	2.81885	4.23461		
**LT (2)**	1.20727	1.19658	1.22473	1.66001	2.82146	1.59827	1.94257	1.90522	1.46225	3.99358	2.78213	4.19124	0.11537	
**LT (3)**	1.14857	1.15048	1.15765	1.82637	3.03073	1.75195	2.13330	2.09159	1.68992	3.95017	2.73909	4.17584	0.21359	0.24987

LL: *L. lamourouxii*; **LO12:** *L. obscura*12; **LO13***:* *L. obscura*13; **LC:** *L. chumphonensis*; **LT:** *L. thailandensis*
